# Quarterly Summary

**Published:** 1854-04

**Authors:** 


### QUARTERLY SUMMARY.

1.—*Treatment of Teeth which have already, or have to be, deprived of their Internal Vitality.*—In the News Letter for January, 1854, Dr. J. F. B. Flagg says, that this is an operation requiring not only much individual experience, but all the information that can be obtained from the experience of others, in order to weigh properly all the circumstances of the case, and to decide correctly with regard to the course most proper to be pursued. The operation of filling the pulp cavity, and not of a tooth, has been performed occasionally for more than fifty years, and with various degrees of success, but this subject has not, until recently, attracted very general attention, and even now, objections to the operation, which must limit its performance, present themselves.

When a tooth is in a condition to require such operation, the patient, as a general thing, has made up his mind to its loss, and when he is led to believe that it may be saved, the whole responsibility of failure rests upon the dentist, unless the matter is so presented to his mind as to enable him to estimate duly the chances of success, and to decide the question himself. Even with those who have been most successful in the performance of this most difficult and tedious of all dental operations, failures do sometimes happen, apart from any oversight as it regards thoroughness in the performance of the operation, there are other circumstances which should be well looked into that must govern the dentist in operating, and in the subsequent treatment should inflammation and alveolar abscess supervene, before he resorts to the removal of the tooth.

The importance and usefulness of the tooth constitutes the first and great consideration, without any reference to the feelings of the patient with regard to the retention of the tooth, who, through fear of extraction, may desire it, as one who would be most likely to shrink from this, would most likely be the one, from peculiarity of temperament, in which he would be least apt to succeed. The general health of the patient and the condition of the tooth should decide the judgment of the operator as to the propriety or impropriety of the operation.

The chances of success are greater in an incisor and cuspidatus than in a bicuspis, and in a bicuspis than a lower molar, and in the latter than in an upper molar, and in a tooth in which it becomes necessary to destroy the pulp than in one in which this is already dead. More thorough preparation and filling are necessary in such cases than in teeth recently deprived of their nerves.* Scrofulous patients, though more liable to abscess, do not usually experience as much pain as persons of a nervous or plethoric habit.

Five hours is long enough for the arsenious acid to remain in a tooth. This is long enough to paralyze the nerve, and in twenty-four hours it may be removed entire with a broach, and when the arsenic only remains this length of time, its deadly influence is not extended beyond what we desire it to act upon.

Dr. Flagg here relates a case in which he had occasion to prepare the roots of the six upper front teeth for artificial substitutes, and after having extirpated the nerves as high up as it was necessary to drill for the pivots, and as he was unable to complete the operation at that time, there being still a little remaining sensation in all the roots, he applied to each a small quantity of arsenious preparation, directing his patient to pick it out the next morning, and wash her mouth with cold water. This she neglected to do, and when he saw her at the expiration of ten days, the action of the arsenic had extended to the surrounding membranes, and destroyed their vitality, so that but a very slight force was necessary to remove the roots.

The future health of the patient should not be risked beyond the worth of a tooth under any circumstances. In forming a diagno-

* "About ten years ago, it was suggested by Dr. Flagg, of Boston, that *previous to filling* such teeth as these, an opening should be made into the nerve chamber by means of a small drill, passing it through the gum about an eighth of an inch below the neck of the tooth, by which a vent is secured, and this new opening protected by means of the gum acting as a valve, thus allowing the gas to escape, and effectually providing against obstructions from foreign substances taken into the mouth. I call attention to this method of treatment in this place for the following reasons :—Firstly, if the operation is carefully performed, it is very generally successful. Secondly, an injustice has been done to Dr. F. in attributing this operation to Dr. Fox and others, of ancient date, when it will readily be perceived that though the operation is the same, the application is very different—one is to cure the disease, the other is to anticipate and thus prevent the disease occurring."

sis, therefore, the idiosyncrasy, present state of the general health of the patient, and amount of suffering induced by the operation, should be properly considered. It would be bad practice to permit a tooth to remain in the mouth after it has extended a morbid action to the antrum maxillæ, or, if in the lower jaw, after suppuration had extended to the external tissues. An approximation to such effects should not be permitted.

2.—*Restoration of Hearing by the insertion of Artificial Teeth.*—Dr. Jas. S. Gilliams states in the Dental News Letter for January, 1854, that the principal function of the *Eustachian tube* appears to be the maintenance of the equilibrium between the air within the tympanum and the external air, thus preventing inordinate tension of the membrane, which would be likely to occur if the pressure were greater on one side than on the other, and the effect of which would be imperfection of audition.

Having laid down this general proposition, the doctor mentions the case of a lady who applied to him to repair a set of artificial teeth, requesting that they should be attended to as soon as possible, as she could not hear when they were out of her mouth. About a year after, his attention was again called to the subject by a lady who called on him for a set of artificial teeth, and at the time was so deaf that it was with great difficulty she could be made to hear. When the teeth were placed in her mouth, she at once exclaimed, accosting Dr. G., "I hear everything you say, I can hear you perfectly well." He has subsequently met with other similar cases where the hearing has been partially restored by the same means.

Dr. W. was led by these observations to inquire whether any change could be induced in the orifice of the Eustachian tube by the excessive approximation of the jaws, whereby the orifice might be closed, or be made to impede the transmission of air through it. Supposing that deafness arose from want of balance between the air within and without the tympanum, or that external deafness was occasioned by causes affecting this membrane, or from obstruction of the external meatus, while the sensibility of the nerve was preserved, the cause of deafness might be more easily understood.

3.—*Dentistry at Kaw Mendi, West Africa.*—Dr. J. Cutler Lefft, writing from the Mendi Mission, says, in Dental News Letter, January, 1854, "I only practice dentistry a little for my associates, and occasionally for the natives, yet I love my old profession, and am pleased to hear of the many improvements which have been made in it from year to year. I have been called, when on business at Freetown, the capital of Sierra Leone, to practice some for the English, German, and Portuguese, while settled there as merchants, consuls, ministers, &c., and a few cases for the wealthy native merchants. If there were a few more Europeans settled at Freetown, it would be a good location for a dental surgeon, but as it is, it would not give a living.

While at Sierra Leone, I have had opportunities to notice the teeth of twenty different tribes, from all parts of Africa. They all have better teeth than the whites, of America, and particularly those from the slave ships. Their change of habits when they come to the colony, many of them becoming servants and cooks in the families of Europeans, where they contract the habit of eating highly seasoned food, and drinking hot tea and coffee, soon show themselves upon their white teeth. Indeed, most of the natives for whom I have operated, are those who have been educated in England. It may be that great use of tobacco is a cause for their soon having bad teeth. Most of them, men and women, smoke very much. Some tribes have a curious custom of filing or cutting either upper or lower incisors, or both, almost to a point, like the South Sea Islanders. They say that they do it to cut "Yanga," i. e. to make them look more beautiful. I have never seen a people prize their teeth more highly than the Kroo tribe, of South Africa. There is no mark of esteem so great to a sweet-heart, as the gift of a tooth. In Sierra Leone, it is an incident of frequent occurrence to meet men of this tribe minus a couple of teeth, i. e. the central superior incisors. Surgeon St. Clair told me an incident which illustrates this point. One of their Kroomen came to him and requested him to extract two beautiful sound incisors. The doctor asked the reason, and was informed by the honest native, that he wanted to send them back to his own country to his sweet-heart.

I had much trouble during one year to keep my instruments in order, free from rust, but I finally avoided the trouble by packing in a common tin box. From my experience, I think surgeons would preserve their instruments by placing them in a tin box or case to shut closely with a trap, not packed with cloth or cotton, &c., but merely in a buckskin, and oiled with mercurial ointment.

4.—*Periostitis.*—Dr. D. B. Whipple states in the News Letter, for January, 1854, that in the treatment of acute periodontitis, all the remedial means which he has been able to employ, occasionally prove unsuccessful; as there seems to be an agent wanting within the pale of the dentist, which will enable him to meet the indications of treatment promptly. Admitting our aid is sought when a case presents in this stage, the object, doubtless, is to produce resolution, and, I believe, to produce this our most effectual means, is depression, and the leech is the agent.

We will suppose a case. A patient applies, for whom a year previous we have treated with great care, the exposed pulp of a central incisor; he now complains of great pain, and, upon examination, find the periosteum in the acute stage; from our diagnosis we direct leeches to be applied. Here the patient passes from our surveillance, and the inference seems to follow that the dentist does not possess all the remedial agents requisite to meet the demand in the treatment of the teeth and their diseases. There is not a dentist who does not wish to possess the power of giving relief, which, in these cases, he is obliged to seek from another. Such wishes have induced me to experiment, and, although my experience has been small, my success induces me to announce it. I think that an artificial substitute for the leech in the local abstraction of blood from the inflamed part about a tooth, can be employed as effectually, something upon the principles of the cupping glass. The dental leech introduced several years ago, is upon this principle, and with it I have treated several cases successfully. The inefficiency complained of by some is attributable to insufficient lancing. The incision must be deep, and my plan is to use the spring lancet, setting it to cut through the integument, and making in some instances a crucial incision. By thus severing the congested blood vessels, a large flow is established, and favoring the determination and relaxation by bathing the part with warm water, I am enabled to obtain as much as by the natural leech. There are other means of meeting such a case, the above is conceded to be the best. All the antiphlogistic remedies may be resorted to, and the counter irritants and discutients, also local depletion meets the case more decidedly. After the leech I direct the part to be bathed with

℞.—Sulph ether, . . ℥ ss
Gum camphor, . . ʒ ss

By the ether we have the anodyne influence and refrigerant also, from its volatile properties, and a tonic action is exerted upon the dilated and relaxed vessels. The camphor, for its discutient properties. (This combination may be used as a prophylactic remedy, when slight vascular excitement is evidenced.) The writer's purpose in this paper is chiefly to describe his mode of using the lancet in conjunction with the artificial leech in the acute stage of periostitis, and the adjunctive treatment he refers to merely from its incidental connection.

5.—*Extraordinary case of Periosteal Irritation during the Eruption of the Dentes Sapientiæ.*—By J. D. White News Letter of January, 1854.—Dr. W. describes the case of a young lady, eighteen years of age, Miss L——, who complained of great pain in all the teeth of both jaws; they were sore to the touch, and very sensitive, particularly the canine teeth; she was unable to chew the softest substance, or brush or rub them with a cloth, and was unable to rinse them with a fluid above or below the temperature of the mouth. The lady was of a sympathetic temperament, great irritability of body, gums pale and rather spongy. The wisdom teeth were not yet erupted. Her medical advisers had been treating her for *neuralgia,* but with no success; they pronounced the roots of the teeth *diseased,* i. e. there were abscesses or fleshy substances growing on the ends, but as the nerves of the teeth were not dead, and all decayed teeth well filled, he knew there could be no alveolar abscess, and therefore pronounced it a case of *irritability of the periosteal membranes, consequent upon the development of the wisdom teeth,* which would pass off when the teeth were erupted. Her physicians did not agree with him, and they consulted another dentist of high standing, who said it was only a case of irritability of the teeth and gums, without any connection with the wisdom teeth, and advised an astringent wash, which, however, afforded no relief. Dr. W. then cut away the gum over the wisdom teeth of one side as an experiment, and the operation was attended with such success as to justify a like operation on the other side. The gums over some of the teeth united in a few months, when the unpleasant symptoms returned, proving conclusively that the first diagnosis was correct; the gums were cut away again over the wisdom teeth, but they closed as before, when they were once more cut and two of the teeth extracted. The gums healed, and all unpleasant symptoms passed off. The first and second inferior and superior molars were still in the mouth on the side from which the two wisdom teeth were removed, which accounted for the fact that the wisdom teeth could not well free themselves from the jaw and gum, whilst on the opposite side the first inferior and first superior molars had been extracted early in life, and the wisdom teeth on this side occupied about the same place as the second molars of the opposite. The jaw was too short to allow of the free eruption of the wisdom teeth without extracting an anterior tooth. This case illustrates many similar, although not so strongly marked.

### CHEMISTRY.

6.—*Alcohol and its Effects on the System.*—Dr. Ducheck has published recently, in the Prager Vierteljahresschrift, a paper on the "Behavior of Alcohol in the Animal Organism," which has been translated for the Philadelphia Medical Examiner. We give a brief abstract of this important article.

There are six varieties of alcohol known: the ethyl, methyl, amyl, acryl, cetyl and glyceryl alcohols. Each of these has an aldehyde which differs from the alcohol by possessing two atoms fewer of hydrogen, and an acid which may be written chemically by adding two atoms of oxygen to the aldehyde.

Alcohol changes into aldehyde by absorbing two equivalents of oxygen, which form two equivalents of water and one of aldehyde. The aldehyde absorbs two more equivalents of oxygen, which in like manner form one atom of water and one of acetic acid. The acetic acid is similarly oxydated into water and formic acid, which changes by the same agency into water and oxalic acid, and this last is ultimately resolved into water and carbonic acid, the last products of the oxydation of alcohol. These metamorphoses will be better understood by the following formulæ.

Alcohol, $C_4 H_6 O_2 + O_2 = 2 H O + C_4 H_4 O_2$ aldehyde.

Aldehyde. $C_4 H_4 O_2 + O_2 = H O + C_4 H_3 O_3$ acetic acid.

Acetic acid, $C_4 H_3 O_3 + O_4 = H O + 2 C_2 H O_3$ formic acid.

Formic acid, $C_2 H O_3 + O = H O + C_2 O_3$ oxalic acid.

Oxalic acid, $C_2 O_3 + O = 2 C O_2$ carbonic acid.

The author, by means of a stomach pump, introduced into the stomachs of three dogs, large quantities of absolute alcohol, and into the rectum of a fourth dog a drachm and a half of fusel oil.

In all four cases, intoxication and death took place. The phenomena were the same, whether the alcohol had been introduced through the stomach or the rectum. The blood was darker, had an alkaline reaction and an aldehyde odor. In two cases aldehyde was detected in the blood, but no further products of the oxydation of alcohol. Alcohol was never found in the blood, and in the stomach only in very small quantities, even when it had been quite recently injected. The urine and the fluid in the ventricles had an odor of ether. No anatomical changes were observed.

It appears, therefore, that alcohol does not exist, even for a short time, as such in the blood. The odor of the breath, which has been relied on by some to establish the circulation of alcohol, is due rather to that alcohol which remains in the cavities of the mouth and throat. Besides this, the bouquet of the liquid, the œnauthic or other ethers which give the peculiar odor to those beverages, communicate their odor to the breath and this is often confounded with the smell of alcohol. The alcoholic smell of the blood, noticed by some anatomists who have made autopsies of drunkards, is really an aldehyde smell, which has been mistaken for the former.

Now where does this oxydation of alcohol to aldehyde take place? Not in the stomach, because it cannot be detected there and because the same results are obtained by injecting the rectum where there is no oxygen to produce the metamorphosis. Not in a remote part of the body as the lungs, for it cannot get there, since alcohol immediately coagulates the blood when it comes in contact with it. The change must therefore take place in the animate vessels, immediately after absorption takes place; or, in other words, the alcohol passes through the mucous membrane, and is immediately converted into aldehyde.

Aldehyde must, therefore, be the intoxicating agent. Experiments upon this matter gave the following results. 1. Aldehyde, introduced into the venous blood or the stomach, produces the same violent symptoms of intoxication as alcohol taken in the ordinary manner. 2. A large quantity of aldehyde suddenly introduced into blood, coagulates it. 3. After the narcotic influence was over, acetic and oxalic acid were found in the blood. These results of the oxydation of aldehyde are never found in the blood of animals dying during the intoxication. Hence it follows not only that aldehyde is the intoxicating agent but that it is eliminated from the system by a process of oxydation. 4. If there is great excess of aldehyde in the system, at a time, it may escape from the lungs with the ordinary exhalations. Hence, in part, the odor of the breath, confounded with the smell of alcohol.

The alternate oxydation, as we have already seen, must be into carbonic acid and water. To determine these excretions, experiments were made upon dogs, taking into account the amount of expired air, the number of respirations and of arterial pulsations and the animal heat. The results were: 1. After taking alcohol, more air is inhaled, showing an increased need of oxygen. 2. In the expired air is found less water and less carbonic acid. 3. The respirations and arterial pulsations, together with the animal heat and consequently the process of oxydation, were greatly augmented.

The blood is deprived of a great amount of oxygen which would otherwise go to the combustion of other substances. The chief constituent of the blood which suffers in this way is the grape sugar, because aldehyde having a greater affinity for oxygen than sugar, the latter remains unconsumed for a time. Now, the proportion of carbonic acid to water produced by the combustion of alcohol is as 1 : 1½, whereas in grape sugar it is as 1 : 1. This accounts for the diminished exhalation of carbonic acid during intoxication. The diminution of the water, is due to the stimulation of the kidneys by the alcoholic liquors injected. The water is diverted to them. Diabetes also takes place, in consequence of the retarded combustion of the sugar.

The further metamorphosis is important. If it is not eliminated as water and carbonic acid, it must be converted into fat. Hence the bloating of drunkards.

The effect upon the nitrogenous constituents of the blood is unknown. Death, when it takes place, is the result of an excess of aldehyde which does not find oxygen enough in the blood to oxydate it to products suitable for elimination.

Experiments upon the chronic effects of alcohol showed that emaciation and tremors of the hind legs were the principal results. In one instance, however, the dog became very fat.

After sudden death from alcohol, no anatomical change was found, except a slight œdema of the lungs. In old drunkards, emaciation was observed, but no change in the brain, not even the thickening of the membrane described by Huss. Contrary to all the statements of anatomists, no change was found in the stomach.

The number of autopsies made is not recorded, and we cannot accept this as a pathological law.

The author abridges his results as follows:

1. Alcohol undergoes, in the system, continual combustion, of which the intermediate products are found in the blood.

2. Intoxication is dependent upon the existence of aldehyde in the blood.

3. The action of aldehyde upon the blood is the rapid withdrawal of its oxygen.

4. Therefore, the combustion of other substances in the blood, and consequently their metamorphoses are prevented or retarded.

7.—*Grape Sugar.*—In Robin and Verdeil's Anatomical Chemistry, there is an excellent *resumé* of our knowledge upon this subject. There are some new facts introduced there in addition to those already in our previous Quarterly Summary.

They sum up the origin of glucose under three heads:

1. The various principles of the body may themselves yield sugar. Animals fed on nothing but meat and bones, had sugar in their liver.

2. Cane sugar disappears, and is transformed into glucose in the liver.

3. When taken as food, it enters the liver directly, and then only can it be found in the portal vein. The cooked amylaceous substances taken as food also are converted into dextrine which is formed into sugar by the liver, and into glucose.

They consider diabetes to be owing to various affections of the lungs, or, perhaps, also to some disease of the medulla oblongata.

Dr. Harley has recently performed some experiments on this subject, which he has reported to the Societé de Biologie, at Paris. They seem to show that the reflex nervous action which results in the formation of sugar, originates in the liver itself, and is occasioned by the stimulating power of the blood of the portal vein on the hepatic branches of the pneumo-gastric nerves. When various stimulating substances were injected into the portal vein, sugar was voided in the urine, and the diabetes persisted for several days. When it is remembered that diabetes often follows the use of alcohol, the importance of this fact will be appreciated, though, as we have already shown, the direct action of aldehyde in the blood, diminishing the regular oxydation of that fluid, is probably the chief cause of this change.

9.—*Use of the Pancreatic Fluid.*—Professor Herbert tied the pancreatic duct in order to determine whether Bernard's opinion in reference to the use of the pancreas was correct. He found that the fatty matters were still emulsified, notwithstanding the non-admission of the pancreatic juice. He also states, contrary to the declaration of the French observers, that the milky hue of the contents of the lacteals is observed above the duct. It will be seen that these results are diametrically opposed to those of Bernard and the committee of the French Academy.

9.—*Rhythmical Movements of the Heart.*—Dr. Brown Sequard attributes the action of the heart to the stimulus of the *carbonic acid* contained in the blood. He bases his opinion upon three principal facts: 1. If warm-blooded animals are prevented from breathing, the beatings of the heart become more frequent for one or two minutes. 2. The hæmadynomometer shows an increase of the circulating energy during asphyxia. 3. All the causes which increase the quantity of carbonic acid in the blood increase the frequency of the pulse.

